# Understanding HPV vaccine preferences among sexual minority men living with HIV in Abuja, Nigeria using a discrete choice experiment

**DOI:** 10.1016/j.vaccine.2026.128771

**Published:** 2026-05-30

**Authors:** Connor R. Volpi, John Chama, Ruxton Adebiyi, Jumoke A. Aigoro, Yerima Jibrin Bawa, Kazeem E. Kolawole, Uchenna Ononaku, Naomi Ayuba, Ashley Shutt, Abayomi Aka, Stephen E. Goldstone, Patrick Dakum, Joel M. Palefsky, Sylvia Adebajo, Karin E. Tobin, Juan Marcos Gonzalez, Rebecca G. Nowak

**Affiliations:** a Johns Hopkins Bloomberg School of Public Health, Baltimore, MD, USA; b Institute of Human Virology, University of Maryland School of Medicine, Baltimore, MD, USA; c Institute of Human Virology Nigeria, Abuja, Nigeria; d International Centre for Advocacy on Rights to Health, Abuja, Nigeria; e Icahn School of Medicine at Mount Sinai, New York, NY, USA; f University of California, San Francisco, San Francisco, CA, USA; g Duke University School of Medicine, Durham, NC, USA

**Keywords:** HPV vaccination, Sexual minority men (SMM), Discrete choice experiment (DCE), Nigeria, Vaccine preferences

## Abstract

**Background:**

Sexual minority men (SMM) in Nigeria face a disproportionate burden of HPV-related diseases, yet HPV vaccination uptake remains limited due to structural, financial, and sociocultural barriers. Understanding SMM’s preferences for vaccination delivery is critical to designing accessible, affirming, and equitable prevention strategies.

**Methods:**

A discrete choice experiment (DCE) was conducted with 250 SMM receiving HIV-related care at an affirming clinic in Abuja, Nigeria. Seven attributes were identified through literature review and stakeholder engagement. Participants completed 12 choice tasks comparing hypothetical HPV vaccination scenarios. Hierarchical Bayes estimation was used to derive individual-level utilities and attribute importance scores.

**Results:**

Participants strongly preferred receiving HPV-related services in SMM-affirming settings and at no cost. Setting type (19.4%) and cost (19.2%) were the most influential attributes, followed by wart prevention (11.4%) and information availability (7.8%). Services described as protecting others from HPV-related outcomes, prevent warts, and require moderate travel (60 min) were moderately preferred. Higher levels of vaccine protection and information availability were less influential in decision-making.

**Conclusions:**

Preferences among SMM in Nigeria emphasize the need for free, affirming, and inclusive HPV vaccination services. Addressing structural barriers, especially stigma and cost, will be essential to increasing vaccine uptake. Findings support targeted, community-informed strategies to reduce HPV disparities and promote health equity.

## 1. Introduction

Human papillomavirus (HPV) is a major public health concern, particularly among sexual minority men (SMM), who experience disproportionately high rates of HPV-related diseases, including anal cancer and genital warts [1,2]. Persistent infection with high-risk HPV types, such as HPV-16 and HPV-18, significantly increases the likelihood of developing anal intraepithelial neoplasia (AIN), a precursor to anal cancer [3]. Although HPV vaccination is a highly effective primary prevention strategy, uptake remains suboptimal in many settings due to structural, informational, and accessibility barriers [4]. This is especially concerning in Nigeria, where HPV-related diseases contribute to a substantial burden of morbidity, yet vaccination efforts remain limited due to systemic healthcare challenges and sociocultural factors that impede vaccine access [5,6].

Although Nigeria initiated a phased nationwide HPV vaccination campaign in 2023, current policy does not include boys, leaving a major gap in coverage [7]. As a result, awareness and uptake remain low among both the general population [8] and key affected groups such as SMM [9]. Although the World Health Organization (WHO) recommends HPV vaccination as a key component of primary prevention [10], access to the vaccine in Nigeria is constrained by factors such as high cost, limited availability, lack of healthcare provider awareness, and stigma associated with sexual health services [5]. The absence of SMM-specific healthcare programs and affirming healthcare environments [11,12] further restricts vaccine access and uptake. As structural and behavioral barriers converge [13], there is an urgent need to understand how SMM in Nigeria perceive and prioritize different aspects of HPV vaccination.

This study used a discrete choice experiment (DCE) to assess HPV vaccine preferences among SMM in Nigeria, identifying key vaccine-related attributes that influence decision-making. The selection of seven attributes was guided by a structured process involving literature review, expert consultations, and stakeholder engagement. Findings from this study will contribute to a deeper understanding of how SMM evaluate HPV vaccination services, informing culturally appropriate strategies to improve vaccine accessibility, enhance healthcare engagement, and reduce HPV-related disparities in this underserved population.

## 2. Materials and methods

A DCE was designed and implemented as part of a survey administered to assess preferences for attributes related to HPV-related interventions [14] among SMM. In DCEs, respondents are asked to make choices that require tradeoffs between aspects (attributes) of experimentally designed healthcare interventions [15]. The frequency with which respondents are willing to accept these tradeoffs help quantify the relative importance of the attributes traded. By presenting participants with realistic, hypothetical scenarios, data from DCEs can be used to estimate the relative importance of characteristics of vaccination programs, such as cost, effectiveness, setting, and convenience [16]. This method has been widely applied in global health to inform the design of equitable, patient-centered programs in low-resource settings [17–19].

The study followed established good-practice guidance to develop and design the DCE [20,21], and to estimate preference weights and attribute importance scores based on participant choices [22,23]. To support the development of the DCE, the study team conducted a literature review, expert consultations, and stakeholder interviews to identify key attributes influencing HPV-vaccination decision-making [14,24]. The literature review focused on HPV vaccination barriers and facilitators among key populations in low- and middle-income countries, particularly in sub-Saharan Africa. Expert consultations included public health professionals, clinicians, and advocates working with SMM in Nigeria, while stakeholder interviews were conducted with community members to ensure the selected attributes reflected lived experiences and context-specific concerns.

**Fig. 1.**
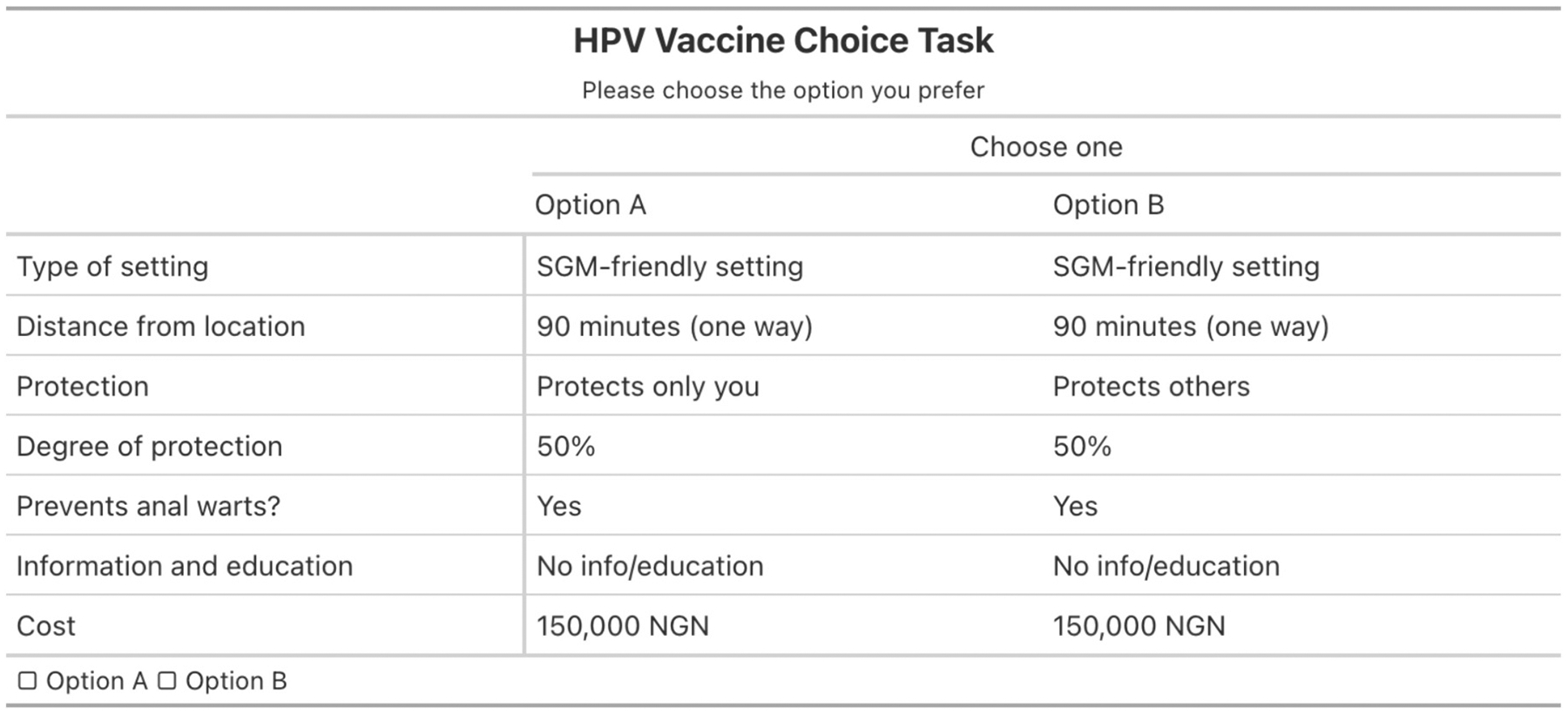
Example choice task.

Seven attributes were selected for inclusion (Table 1). These attributes represented general aspects associated with vaccination programs. A series of levels were also selected to describe the quality or performance of programs under each attribute. Cost levels were selected based on a combination of factors, including published and locally available HPV vaccine pricing information in Nigeria, stakeholder input regarding perceived affordability among SMM, and the need to capture a realistic range of potential out-of-pocket costs within the local healthcare context. The selected levels were intended to reflect meaningful differences between low-, moderate-, and high-cost vaccination scenarios that participants may encounter in practice. Each DCE task presented two alternatives, allowing participants to select their more preferred option. (Fig. 1). The example shown in Fig. 1 is illustrative only; across the full set of DCE tasks, multiple attribute levels varied simultaneously between alternatives. A balanced overlap design with no imposed prohibitions was used to populate attribute combinations, ensuring all level combinations were plausible. Each participant completed 12 choice tasks, optimizing preference estimation while minimizing cognitive burden [21].

Eligible participants were adults aged 18 years or older who were living with HIV, assigned male sex at birth, and reported a history of anal sex with another man. Participants were recruited while visiting the TRUST/ICARH clinic in Abuja, a healthcare facility known for providing SMM-friendly services in a supportive and nonjudgmental environment. TRUST/ICARH is a community-based clinic in Abuja that provides comprehensive and confidential HIV care, including antiretroviral therapy (ART) services, tailored to the needs of SMM. As a result, most participants were already engaged in HIV-related services at the clinic, making it a relevant setting for exploring preferences around HPV vaccination.

A Random-Parameters Logit (RPL) model following a Hierarchical Bayes (HB) estimation method was used to link the choices made by each respondent and the differences in attribute levels across alternatives. Effect coding was employed for categorical attribute levels in the model specification. The analysis produced expected preference weights or marginal utility of attribute levels, which are log-odds indicating how the probability of choice of a vaccination program changed when the program included a specific attribute level, all else equal. Mean estimates accounted for systematic differences in preferences across respondents. From the expected preference weights, we calculated attribute importance as the maximum difference in preference weights across levels in an attribute normalized to add up to one [23]. Thus, these importance values provide a quantitative measure of each attribute’s maximum influence on decision-making between vaccination programs given the levels chosen for the attributes. All analyses were performed using Sawtooth Software SSI Web (Sawtooth Software, Provo, UT, USA) for conjoint modeling [25].

Subgroup analyses were conducted using individual-level part-worth utilities derived from hierarchical Bayes estimation. Mean utilities for selected attribute levels were compared across age groups using linear regression models. Willingness-to-pay (WTP) estimates were calculated by taking the ratio of mean attribute utilities to the mean cost utility and scaling by the highest cost level (NGN 300,000), consistent with standard approaches in discrete choice experiments [26]. Age was selected for subgroup analysis given its relevance to HPV risk and vaccination decision-making, and to maintain interpretability given sample size considerations. Ethical approval for this study was obtained from the institutional review boards of the University of Maryland, Baltimore; Johns Hopkins University; and the National Health Research Ethics Committee of Nigeria (NHREC). Written informed consent was obtained from all participants prior to enrollment.

## 3. Results

Table 2 presents the demographic and behavioral characteristics of 250 SMM. The sample was evenly split between ages ≤29 and ≥30, with 44.2% completing senior secondary education and 79.5% preferring English. Most participants were not formally employed at the time of the survey (65.3%); however, 60.8% identified their occupation category as working professional. Bisexual identity was most common (47.4%), and the majority identified as men (94.8%) and were unmarried (80.3%). Nearly all participants received care at TRUST/ICARH (94.0%) and 85.8% felt uncomfortable receiving care elsewhere. The median travel time to the clinic was 60 min (Interquartile Range [IQR]: 45–70). Over half (52.2%) were virally suppressed, while 57.3% reported condomless anal sex and a median of 5 male partners in the past six months (IQR: 3–8). Tobacco use (51.6%) and party drug use (36.6%) were also notable. The median duration of living with HIV was 5 years.

**Fig. 2.**
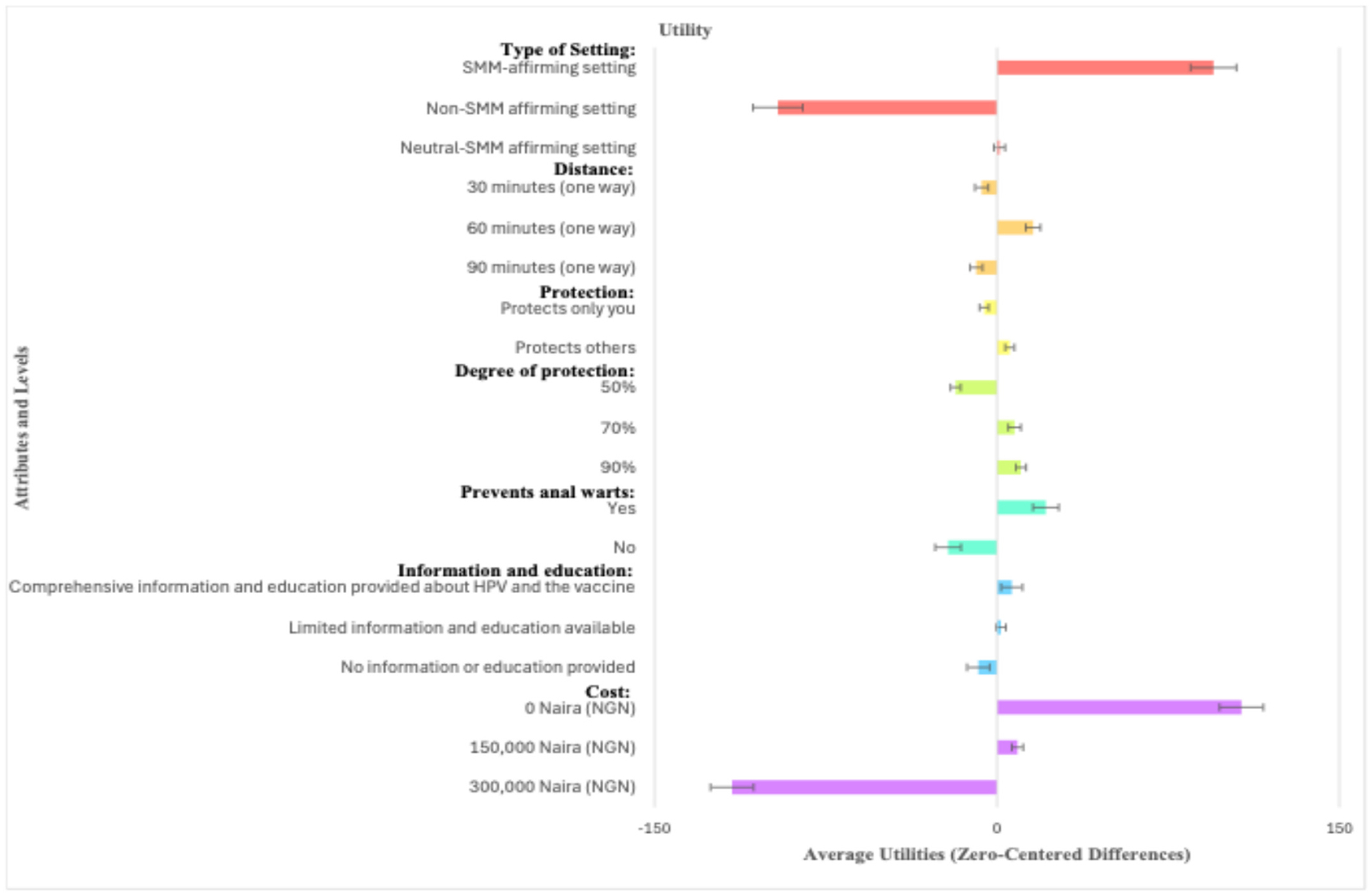
Average utilities for vaccine attribute levels from discrete choice experiment among sexual minority men in Nigeria.

Table 3 presents the results from the DCE using a random parameter logit model. Participants showed relatively strong preferences for receiving HPV-related services in SMM-affirming settings (β = 0.67, 95% CI: 0.59–0.76, *p* ≤ 0.001) and were relatively averse to non-SMM-affirming settings (β = −0.67, 95% CI: −0.75 to −0.59, p ≤ 0.001). In terms of cost, free services (₦0) were significantly preferred (β = 0.708, 95% CI: 0.62–0.79, p ≤ 0.001), with decreasing utility as cost increased. Services priced at ₦300,000 were relatively disfavored (β = −0.824, 95% CI: −0.91 to −0.74, p ≤ 0.001). Significant preference heterogeneity was observed across multiple attributes. Fig. 2 presents the average zero-centered utility estimates for each attribute level included in the DCE. The figure visually illustrates the direction and magnitude of participant mean preferences for vaccine delivery attributes, with confidence intervals representing the standard errors around each estimate.

Participants strongly preferred receiving HPV-related services in SMM-affirming settings, which yielded the highest positive utility, while non-SMM affirming settings were significantly disfavored, with one of the most negative utility scores. The neutral setting had no discernible influence on preferences, clustering around the zero-utility line. Cost was a key driver of preferences. As expected, receiving the vaccine at no cost (0 Naira) was strongly preferred, whereas increasing the cost to ₦300,000 (₦ = Naira) was associated with the most pronounced reduction in preference weights observed in the data. A moderate cost of ₦150,000 yielded a small positive utility but was far less preferred than the no-cost option.

Participants preferred services that prevent anal warts, which had a higher preference weight than no protection. Additionally, the average patient favored options that protected others over those that only protect the individual, reflecting a potential altruistic concern in decision-making. Preference-weight estimates for distance showed that participants had a moderate preference for traveling 60 min one way compared to 30 or 90 min, which yielded near-zero or slightly negative utilities.

Regarding the degree of protection, 50% protection had the lowest preference weight for the attribute, indicating lower acceptability. However, 70% and 90% protection levels had indistinguishable preference weights, suggesting that incremental increases in protection above 50% were not strongly differentiated by the average respondent. Finally, the information and education attribute did not significantly influence preferences. All levels—comprehensive information, limited information, and no information—clustered near zero utility, indicating limited importance in shaping choice behavior within this sample, although the “no information” level was weakly disfavored.

Attribute relative importance showed that type of setting (19.4%) and cost (19.2%) were the most influential factors in decision-making, followed by prevention of anal warts (11.4%), information and education (7.8%), distance from location (4.4%), degree of protection (4.0%), and protection of others (3.4%). Participants preferred services that prevent anal warts (β = 0.129, 95% CI: 0.08–0.18, *p* ≤ 0.001) over those that do not (β = −0.129, 95% CI: −0.18 to −0.08, p ≤ 0.001). Distance also influenced vaccination preferences, with a 60-min one-way travel being significantly more preferred (β = 0.15, 95% CI: 0.07–0.23, *p* = 0.0003), while 30- and 90-min distances were not statistically significant at the 5% level.

Regarding protection, participants preferred options that protect others (β = 0.08, 95% CI: 0.03–0.13, *p* = 0.0012) over those that only protect themselves (β = −0.08, 95% CI: −0.13 to −0.03, p = 0.0012). For degree of protection, 50% protection was significantly less preferred (β = −0.10, 95% CI: −0.18 to −0.02, *p* = 0.0101), while 70% and 90% did not show statistically significant differences. Although participants tended to prefer having some level of information and education, none of the differences between information levels were statistically significant at the 5% level. Nevertheless, having no information or education available showed a marginal negative trend (β = −0.065, *p* = 0.086).

To further explore heterogeneity in preferences, subgroup analyses using individual-level hierarchical Bayes utilities were conducted (Table 4). Preferences were broadly consistent across age groups. No statistically significant differences were observed for efficacy, cost, service setting, or travel time (all *p* > 0.20), although older participants showed a non-significant trend toward greater cost sensitivity. Willingness-to-pay (WTP) estimates indicated that participants were willing to pay approximately NGN 26,000 ($ ~ 18 USD) for higher efficacy (90%) and NGN 249,000 ($ ~ 182 USD) for care in an SMM-affirming setting.

## 4. Discussion

This study provides critical insights into the preferences and decision-making factors that may influence HPV vaccination uptake among SMM in Nigeria. Using a DCE, we identified key attributes that may drive engagement with HPV vaccination services among SMM in Nigeria. Our findings underscore the importance of cost and affirming healthcare environments in shaping preferences, with affordability emerging as the most influential factor. Given the limited availability of HPV vaccines for boys and screening programs in Nigeria, these results highlight the significant role that financial barriers play in healthcare access among SMM [27,28]. This aligns with prior research indicating that out-of-pocket costs remain a major deterrent to healthcare utilization in low- and middle-income countries, particularly for marginalized populations [29,30].

Another key finding was the strong preference for receiving care in SMM-affirming settings, with non-affirming environments being the most strongly disfavored. This preference reflects the broader structural barriers that SMM face when accessing healthcare in Nigeria, including stigma, discrimination, and fear of disclosure [31,32]. The lack of inclusive healthcare services may deter individuals from seeking HPV vaccination or screening by fostering fear of discrimination, mistrust of providers, and concerns about confidentiality, barriers that can discourage open dialogue and consistent engagement with preventive care [33]. As previous studies have demonstrated, stigma-related barriers significantly contribute to health disparities among SMM [34], necessitating the implementation of culturally competent, community-centered healthcare initiatives to improve engagement with preventive services [35].

Our results also suggest that clinical effectiveness, including wart prevention and degree of protection, plays a moderate role in decision-making, but is secondary to cost and healthcare setting. This finding suggests that while SMM value the health benefits of HPV-related interventions, logistical and structural barriers may outweigh clinical considerations when making healthcare decisions. As outlined in the Health Equity Promotion Model [36], access to care among sexual minority populations is shaped by intersecting structural and environmental contexts—including social exclusion, discrimination, and institutional stigma—that can constrain individuals’ ability to prioritize long-term health outcomes. These systemic barriers can make even evidence-based preventive interventions, like HPV vaccination, less accessible or appealing despite recognized health benefits. Similarly, information and education about HPV, while important, were not as influential as cost or healthcare setting. These results highlight the need for multi-level interventions [37,38] that address affordability and accessibility while also integrating targeted educational initiatives to enhance HPV awareness and vaccine acceptability.

Interestingly, travel distance had a relatively low importance score, with participants showing a slight preference for moderate travel times over shorter or longer commutes. This finding may reflect a desire to seek care in locations outside of one’s immediate community, where greater anonymity and reduced risk of being recognized can help protect privacy and queer identity. As research on LGBTQ mobilities has shown [39], individuals often alter their travel routes or extend their commute in order to avoid discrimination, reduce visibility, and feel safer while accessing public services, especially in environments perceived as hostile or non-affirming. This may suggest that SMM are willing to travel for services if they perceive them as high quality, inclusive, or more affordable.

The substantial willingness-to-pay for affirming healthcare settings may also help explain why participants appeared willing to tolerate moderate travel times, suggesting that SMM may strategically navigate healthcare geographies in search of spaces that offer greater safety, anonymity, and social affirmation. This finding aligns with emerging literature on queer mobility and “therapeutic landscapes,” in which marginalized populations travel beyond their immediate communities to access trusted and identity-affirming care environments [40–42]. This finding may have implications for the design of HPV prevention programs, suggesting that centralized services—particularly those perceived as affirming and trustworthy—could be more appealing to SMM than widely dispersed, non-specialized clinics. However, further research is needed to assess whether centralized models enhance trust and engagement in this context.

This study has several limitations. First, although the DCE methodology allows for rigorous estimation of preferences, it relies on hypothetical scenarios that may not fully reflect real-world decision-making constraints or behaviors [15]. Second, there is the potential for selection bias [43], as individuals who chose to participate in a study focused on HPV-related healthcare preferences may differ systematically from those who did not, possibly having greater awareness of or access to care. Additionally, because participants were recruited exclusively from an SMM-affirming HIV care clinic, preferences for affirming healthcare settings may have been amplified relative to SMM not currently engaged in affirming care environments or those disconnected from healthcare services altogether. Third, despite efforts to reduce cognitive burden through careful design and piloting, the complexity of the choice tasks may still have influenced how participants made decisions. Additionally, the discrete choice experiment did not account for the number of required vaccine doses—an important consideration, particularly for individuals living with HIV who are recommended to receive three doses [44].

Finally, an additional limitation of the study is that participants were asked about prevention of genital warts rather than cancer specifically, which may influence the interpretation of prevention-related responses. Additionally, the “protection” attribute may have been interpreted differently across participants. Some participants may have understood protection as directly protecting sexual partners from HPV-related outcomes, rather than indirectly reducing transmission risk through vaccination, which could have influenced preference estimates related to this attribute.

Future research should incorporate these attributes to better understand preferences around follow-up and continuity of care. Despite these limitations, this study provides novel and contextually grounded insights into HPV vaccine preferences among sexual minority men in Nigeria, offering valuable evidence to inform the development of affirming and accessible vaccine delivery strategies in similar settings.

## 5. Conclusions

Addressing HPV-related disparities among SMM in Nigeria requires a multifaceted approach that prioritizes affordability, inclusive healthcare environments, and accessible, evidence-based education. Our findings emphasize that reducing financial barriers and expanding SMM-affirming services are key strategies for increasing HPV vaccine uptake and engagement with preventive care. Future public health initiatives should integrate subsidized or government-supported HPV vaccination programs, promote training for healthcare providers to deliver nonjudgmental care, and develop targeted outreach efforts that address both cost concerns and informational gaps. By aligning HPV prevention strategies with the preferences of SMM, policymakers and healthcare stakeholders can design more effective, community-centered interventions to reduce HPV-related disease burden and improve health equity in Nigeria.

## Figures and Tables

**Table 1:** HPV vaccine attributes and levels.

Attribute	Participant-friendly label and description	Levels
Type of setting	Label: Vaccination Environment Description: What type of environment would you prefer for receiving the HPV vaccine?	SMM-friendly setting Non-SMM friendly setting Neutral-SMM friendly setting
Distance from location	Label: Distance to Vaccination Site Description: How far are you willing to travel to receive the HPV vaccine?	30 min (one way) 60 min (one way) 90 min (one way)
Protection	Label: Type of Protection Description: Who benefits from the protection provided by the HPV vaccine?	Protects only you Protects others
Degree of protection	Label: Degree of Protection Description: How effective is the HPV vaccine in protecting against HPV-related health issues?	50% 70% 90%
Prevents anal warts?	Label: Prevention of Anal Warts Description: Does the HPV vaccine prevent anal warts?	Yes No
Information and education	Label: Information and Education Description: How much information and education about HPV and the vaccine is provided?	Comprehensive information and education provided about HPV and the vaccine Limited information and education available No information or education provided
Cost	Label: Cost of Vaccination Description: How much will the HPV vaccine cost you?	₦0 (NGN) ₦150,000 (NGN) N300,000 (NGN)

**Table 2:** Demographic and behavioral distributions of SMM.

*Characteristic:*	Total (*n* = 250)
	N (%)
*Age:*	
≤29	130 (52.2)
≥30	119 (47.8)
*Highest level of education:*	
Junior Secondary	50 (20.1)
Senior Secondary	110 (44.2)
Higher than Senior Secondary	89 (35.8)
*Preferred language:*	
English	198 (79.5)
Hausa	51 (20.5)
*Currently employed?*	
No	162 (65.3)
Yes	86 (34.7)
*Occupation?*	
Not Working	33 (13.5)
Student	63 (25.7)
Working	149 (60.8)
*Monthly income (in Naira):*	
Refusal	38 (15.3)
<70,000	108 (43.4)
≥70,000	103 (41.4)
*Sexual orientation:*	
Gay	56 (22.5)
Bisexual	118 (47.4)
Queer	75 (30.1)
*Gender identity:*	
Man	236 (94.8)
Woman	0 (0.0)
Both	13 (5.2)
*Marital status:*	
Married	49 (19.7)
Not Married	200 (80.3)
*Receiving care at TRUST/ICARH?*	
No	15 (6.0)
Yes	234 (94.0)
*Would you feel comfortable receiving care in a different setting?*	
No	199 (85.8)
Yes	33 (14.2)
*Distance from clinic, in minutes, one way (Median, [IQR]*	60 (45–70)
*Are you virally suppressed (<20 copies/ml)?*	
Don't know	67 (26.9)
No	52 (20.9)
Yes	130 (52.2)
*Anal sex without a condom in the last month?*	
No	105 (42.7)
Yes	141 (57.3)
*Number of male partners in the last month (Median, [IQR])*	5 (3–8)
*Currently smoke tobacco?*	
No	120 (48.4)
Yes	128 (51.6)
*Use party drugs?*	
No	156 (63.4)
Yes	90 (36.6)
*Years living with HIV*	
≤5 years	144 (57.8)
≥6 years	105 (42.2)

Abbreviations: HIV, human immunodeficiency virus; ICARH, International Centre for Advocacy on Rights to Health; IQR, interquartile range; ml, milliliters.

**Table 3:** Random parameter logit model results.

Attributes	Coefficient (SE)	95% CI	*p*-value	Relative importance (%)
Type of setting:				19.4
SMM-affirming setting	0.67 (0.041)	(0.59–0.76)	**≤0.001**	
Non-SMM affirming setting	−0.67 (0.040)	(−0.75 to −0.59)	**≤0.001**	
Neutral-SMM affirming setting	−0.00 (0.040)	(−0.08–0.08)	0.997	
Distance from location:				4.4
30 min (one-way)	−0.07 (0.040)	(−0.15–0.00)	0.060	
60 min (one-way)	0.15 (0.042)	(0.07–0.23)	**0.0003**	
90 min (one way)	−0.08 (0.041)	(−0.15–0.00)	0.067	
Protection:				3.4
Protects only you	−0.08 (0.024)	(−0.13 to −0.03)	**0.0012**	
Protects others	0.08 (0.024)	(0.03–0.13)	**0.0012**	
Degree of protection:				4.0
50%	−0.10 (0.039)	(−0.18 to −0.02)	**0.0101**	
70%	0.06 (0.042)	(−0.02 to 0.14)	0.139	
90%	0.04 (0.039)	(−0.04–0.12)	0.319	
Prevents anal warts:				11.4
Yes	0.129 (0.025)	(0.08–0.18)	**≤0.001**	
No	−0.129 (0.025)	(−0.18 to −0.08)	**≤0.001**	
Information and education:				7.8
Comprehensive information and education provided about HPV and the vaccine	0.034 (0.039)	(−0.04–0.11)	0.385	
Little information and education available	0.032 (0.040)	(−0.04 to 0.11)	0.431	
No information or education provided	−0.065 (0.038)	(−0.14 to 0.00)	0.086	
Cost:				19.2
₦0 (NGN)	0.708 (0.045)	(0.62–0.79)	**≤0.001**	
₦150,000 (NGN)	0.116 (0.037)	0.04–0.19)	**0.0018**	
₦300,000 (NGN)	−0.824 (0.043)	(−0.91 to −0.74)	**≤0.001**	

**Table 4:** Subgroup differences in preferences by age using individual-level hierarchical Bayes utilities.

Attribute (level)	≤29 (Mean utility)	≥30 (Mean utility)	Difference (β)	p-value
90% efficacy	0.339	0.329	−0.010	0.861
High cost	−3.613	−4.058	−0.445	0.209
SMM-friendly	3.074	3.275	+0.202	0.577
90 min travel	−0.157	−0.203	−0.046	0.492

## Data Availability

Data will be made available on request.
